# Sex Differences in Arthritis, Pain, and Frailty Among Mexican American Older Adults

**DOI:** 10.1093/geroni/igaf122.3843

**Published:** 2025-12-31

**Authors:** Jamil Okada, Soham Al Snih, Marielle Rominger

**Affiliations:** UTMB, Galveston, Texas, United States; The University of Texas Medical Branch at Galveston, Texas, Galveston, Texas, United States; Dell Medical School, Austin, Texas, United States

## Abstract

Frailty is a significant public health concern in older adults because of its associated increased risk of falls, disability, cognitive decline, decreased quality of life, and mortality. This study investigated sex differences in the relationship between arthritis and pain with frailty over 21-years of follow-up among non-frail at baseline Mexican American older adults. Participants (N = 1632) were from the Hispanic Established Population for the Epidemiologic Study of the Elderly. Measures included socio-demographics and health characteristics. Independent variables were self-reported physician diagnosed arthritis and pain on weight bearing. Frailty (outcome variable) was defined as ≥ 3 criteria: unintentional weight loss of > 10 pounds, weakness, exhaustion, low physical activity, and slowness. Participants were divided into four groups: no arthritis-no pain, pain-no arthritis, arthritis-no pain, and arthritis-pain. Generalized estimating equation models were used to estimate the odds ratio (OR) and 95% confidence interval (95% CI) of frailty as a function of arthritis and pain over time by sex, controlling for covariates. Among male participants, those with pain only or arthritis and pain had greater (OR = 2.53, 95% CI = 1.50-4.25 and OR = 2.33, 95% CI = 1.06-5.12, respectively) odds of frailty over time than those without pain and without arthritis after controlling for covariates. Among female participants, those with pain only had greater (OR = 1.82, 95% CI = 1.15-2.88) odds of frailty over time than those without arthritis and without pain after controlling for covariates. Pain was a significantly associated with frailty over time in both males and females. However, arthritis and pain were significantly associated with frailty over time in male participants only.

